# 

**Published:** 1878-04

**Authors:** 


### BOOKS AND PAMPHLETS RECEIVED.

A Manual of Nursing; prepared for the Training School for Nurses attached to Bellevue Hospital. New York: G. P. Putnam's Sons. 1878. 12 mo.; flexible cloth, pp. 143. Price $1.00.

St. Bartholomew's Hospital Reports. Edited by W. S. Church, M. D., and Alfred Wallett, F. R. C. S. Vol. XIII. London: Smith, Elder & Co. 1877. 8vo.; cloth, pp. 354.

A Practical Manual of the Diseases of Children, with a Formulary. By Edward Ellis, M. D., etc. Third edition. Philadelphia: Lindsay & Blakiston. 1878. 8vo.; cloth, pp. 385. Price $2.50.

Injuries to the Eye and their Medico-Legal Aspect. By Ferdinand von Arlt, M. D., etc. Translated, with the permission of the author, by Chas. S. Turnbull, M. D., etc. Philadelphia: Claxton, Remsen & Haffelfinger. 1878. 8vo.; cloth, pp. 198.

Cerebral Hyperæmia the Result of Mental Strain or Emotional Disturbance. By William A. Hammond, M. D., etc. Read before the New York Neurological Society (in outline), Nov. 5th, 1877. New York: G. P. Putnam's Sons. 1878. 12mo.; flexible cloth, pp. 108. Price $1.00.

What Am I? A Valedictory Address to the Graduates, delivered at the close of the Forty-first Session of the Medical Department of the University of Louisville, Feb. 28, 1878. By J. M. Bodine, M. D., etc. Published by request.

Are Eczema and Psoriasis Local Diseases of the Skin, or are they Manifestations of Constitutional Disorders? By L. Duncan Bulkley, A. M., M. D. Ext. Trans. International Med. Congress. Phila., Sept., 1876.

On the Recognition and Management of the Gouty State of Diseases of the Skin. By L. Duncan Bulkley, A. M., M. D. Reprint *Amer. Practitioner* Nov., 1877.

On the So-called Eczema Marginatum of Hebra (Tinea Circinata Cruris) as observed in America. A Clinical Study. By L. Duncan Bulkley, A. M., M. D. Read at the first annual meeting of the American Dermatological Association, Niagara Falls, Sept. 4, 1877. Reprint from the CHICAGO MEDICAL JOURNAL AND EXAMINER, Nov., 1877.

Bathing, Cupping, Electricity, Massage. A Comparison of the Therapeutic Effects of Bathing, or Cupping, or Atmospheric Exhaustion of Electricity in the Form of Galvanism and Faradism, and of Massage, in the Treatment of Debilities, Deformaties and Chronic Disease. By David Prince, M. D., of Jacksonville, Ill. Reprint *Amer. Pract.*, Feb., 1878.

Surgical Uses, other than Hæmostatic of the Strong Elastic Bandage. By Henry A. Martin, M. D., etc. Reprint from the transactions of the Amer. Med. Ass'n. for 1877.

Report on Heating and Ventilation. Prepared for the Trustees of the Johns Hopkins Hospital, Baltimore, Md. By John S. Billings, Surgeon U. S. A., 1878.

Report of the Department of Health of the City of Chicago for the year 1877.

---

BORAX AND NITRATE OF POTASSIUM IN SUDDEN HOARSENESS.—These two salts have been employed with advantage in cases of hoarseness and aphonia occurring suddenly from the action of cold (" La France Medicale"). The remedy is recommended to singers and orators whose voices suddenly become lost, but which by this means can be recovered almost instantly. A little piece of borax the size of a pea is to be slowly dissolved in the mouth ten minutes before singing or speaking; the remedy provokes an abundant secretion of saliva, which moistens the mouth and throat. This local action of borax should be aided by an equal dose of nitrate of potassium, taken in a warm solution before going to bed.—*Philadelphia Times.*

### ANNOUNCEMENTS FOR THE MONTH.

### SOCIETY MEETINGS.

Chicago Medical Society—Mondays, April 1 and 15.

Chicago Society of Physicians and Surgeons—Mondays, April 8 and 22.

### CLINICS.

MONDAY.

Eye and Ear Infirmary—2 to 4 p. m., by Prof. Holmes and Dr. Hotz—2 p. m., Prof. Jones.

Mercy Hospital—2 to 3 p. m. Surgical, by Prof. Andrews.

Rush Medical College—1:30 p. m. Medical, by Dr. Bridge; 2:30 p. m. Dermatological and Venereal, by Dr. Hyde.

County Hospital—8 p. m. Necropsy, by Dr. Danforth.

Woman's Medical College—3 p. m. Surgical, by Prof. Owens.

TUESDAY.

County Hospital—1:30 p. m. Medical, by Prof. Lyman; 2:30 p. m. Surgical, by Prof. Parkes.

Mercy Hospital—2 p. m. Medical, by Prof. Hollister.

Eye and Ear Infirmary—2 p. m. Prof. Jones.

WEDNESDAY.

County Hospital—1:30 p. m. Ophthalmological, by Dr. Montgomery. 2:30 p. m. Gynecological, by Dr. Bridge.

Mercy Hospital—2 p. m. Eye and Ear, by Prof. Jones.

Rush Medical College—4 p. m. Diseases of the Chest, by Prof. Ross.

THURSDAY.

Mercy Hospital—2 p. m. Medical, by Prof. Davis.

Rush Medical College—1:30 p. m. Neurological, by Prof. Lyman.

Eye and Ear Infirmary—2 to 4 p. m. Operations by Prof. Holmes and Dr. Hotz.

FRIDAY.

Mercy Hospital—2 p. m. Medical, by Prof. Davis.

County Hospital—1:30 p. m. Medical, by Prof. Quine; 2:30 p. m., Surgical, by Prof. Powell.

Woman's Medical College—10 p. m. Ophthalmological, by Dr. Montgomery.

SATURDAY.

Rush Medical College—2 p. m. Surgical, Prof. Gunn.

Chicago Medical College—2 p. m. Surgical, by Prof. Andrews and Isham; 3 p. m., Diseases of the Chest, by Prof. Johnson.

Woman's Medical College—12 m. Gynecological, by Prof. Fitch; 3 p. m. Dermatological, Dr. Maynard.

Special Clinics daily, from 2 to 4 p. m., at the South Side Dispensary, and at the Central Free Dispensary.

For schedule of lectures at the colleges, apply to the college janitors.

